# Knowledge, attitude and practice of physical exercises among pregnant women attending prenatal care clinics of public health institutions in Hawassa city, Sidama, Ethiopia, in 2021: descriptive cross-sectional study

**DOI:** 10.1186/s12905-023-02756-8

**Published:** 2023-11-27

**Authors:** Berhan Tsegaye Negash, Yitateku Alelgn

**Affiliations:** Department of Midwifery, Collage of Medicine and Health Science, Hawassa University, Hawassa, Ethiopia

**Keywords:** Pregnancy, Physical exercise, Knowledge, Attitudes, Practice

## Abstract

**Background:**

Participating in physical exercise enhances the physical and mental health of pregnant women. Preventing excessive weight gain, decreasing gestational hypertension, reducing back pain and labor complications are some of the main benefits of physical exercise during pregnancy and childbirth. Scrutinizing factors associated with sedentary life style among women during pregnancy could aid to design effective strategies to tackle the problem. Despite its benefit, little is explored about knowledge, attitude and practice of physical exercise among pregnant women in the study setting.

**Objective:**

To assess prevalence of knowledge, attitude and practice of physical exercise among pregnant women who visit antenatal care at public health facilities of Hawassa town, Ethiopia, in 2023.

**Methods:**

Facility based survey was conducted from November-December, in 2021. Data were collected using interview administered and structured questionnaire. Data were cleaned, coded and entered using Epi-data 4.6 and exported into SPSS 25 for analysis. Descriptive statistics was done using frequency count, percentage and mean values of variables. Finally, findings are presented using text, tables and charts.

**Results:**

All of the study subjects completed interview making a response rate of 100% in this study. The mean adequate knowledge score was 42.2%. Positive attitude towards physical exercise during pregnancy was accounted as 63.7% and proportion of good practice of physical exercise was as 35.8%. Regarding practice of exercise, most (95.9%) of the subjects walk, however; only 11(8.9%) women perform pelvic floor exercise were the highest and least practiced physical exercise. Concerning knowledge of exercise, prevent excess weight 72.1% and increasing energy 53.2% were the commonly known benefits of physical exercise. Breathing difficulty (41.3%), chest pain (39.8%) and premature labor (34.0%) were the predominant perceptions of contra-indication of physical exercise during pregnancy.

**Conclusions:**

In conclusion, the proportion of knowledge, attitude and practice of antenatal exercise is found to be sub-optimum in the study area. Therefore, health education should be enhanced about the benefit of physical exercise during pregnancy.

## Background

Pregnant women should be encouraged to keep up work out routines as a chance to enjoy them. Antepartum physical exercises are designed to help pregnant women and their growing fetus healthy [1, 2]. However, sedentary behavior before or during pregnancy is usually associated with poor maternal and newborn outcomes [3]. Moreover, it has long term impact like chronic diseases, increased blood pressure, blood sugar, overweight and deaths [9].

World Health Organization (WHO) reported that physical exercise is the fourth leading causes of mortality [4].

Physical exercise is defined as a planned, structured, and repetitive subset of physical activity that maintains physical fitness and well-being. The world confederation for physical therapy exercise enables pregnant women to develop, maintain and restore maximum movement and functional ability throughout pregnancy [1]. Regular physical exercise is recommended for the benefit of population the overall health [15, 16]. Naturally, in the absence of medical or obstetric complications, low and moderate intensity aerobic exercise is recommended during pregnancy. Meanwhile, pregnant women should be restricted from activities which increase the risk of falls or contacts [5, 6].

Physical exercise has both physical and mental advantages for pregnant woman and their growing fetus. Physically, it prevents excessive weight gain; reduces the risk of medical complications, minimize the incidence of macrosomia, prevents lower back pain [7–9], decreases the risk of pregnancy complications, prevent intrauterine fetal growth restriction, and reduce urinary incontinency [3, 4]. Mentally, it improves sleeping pattern and improve health perception [2]. According to American College of Obstetrics and Gynecology standards (ACOG) estimation, most (79%) of pregnant women attain sedentary life [7–9].

According to the National Institute for Health and Care Excellence (NICE) and ACOG guidelines some modification of exercise is necessary depending on the needs of the mother and the fetus. For example, ACOG advised women to engage in low or moderate impact exercises for 30 min to prevent excessive weight gain and improves their mental health [10]. Although physical exercise has no proven health hazards, the lifestyle of pregnant women remains the same throughout pregnancy [11].

Knowledge is considered an essential precursor for behavior change processed [14]. Pregnant women tend to demonstrate a lack of knowledge regarding physical activity during pregnancy [15, 16]. Based on the report of previous study, women who were given guidelines for physical exercise during pregnancy reported that they did physical exercise following these guidelines [17]. Relatively low proportion of pregnant women reported on adherence to exercise interventions from health providers during pregnancy [18]. Knowledge of women about benefits and risks of antenatal exercise was estimated as 19% in Zambia [13], Nigeria (46.6%) [14]. A study conducted in Northwest Ethiopia indicates that one out of five (39.5%) women had suboptimum knowledge about physical exercise during pregnancy [15].

Regarding about actual practice of exercise among women during pregnancy, nearly 40% of women in Brazil [10] and 35.8% women in Saudi Arabia [11] practiced physical exercise during pregnancy. In Ethiopia, almost half (48.5%) of pregnant women has reported performing physical exercise in Tigray [12].

Attitude of pregnant women towards physical exercise is affecting their practice during pregnancy. For example, a common misconception and concern among pregnant women, families, and some obstetricians is exercise during pregnancy can lead to miscarriage, poor fetal growth, musculo-skeletal pain, musculoskeletal damage and premature birth [11, 15].

A study in Pakistan indicates that 87.2% of pregnant women had poor attitude toward exercise in pregnancy [16] and more than half (55.3%) of women in Northwest Ethiopia had negative attitude towards exercise during pregnancy [15]. For instance, some women decide to continue their physical exercise throughout pregnancy because it is widely believed that it is safe for the developing fetus and beneficial for overall health and wellness. The majority of pregnant women, according to the research, do not exercise in any way and tend to engage in fewer physical activities overall, including chores and work-related tasks [12, 13].

Globally, the concept of a fit pregnancy has steadily grown in popularity due to the great results it has produced over the past 20 years [14, 15]. In developing countries, cultural acknowledgment, ethnic practices, beliefs, maternal age, unwanted pregnancy, level of education of the women, health care access, availability of trained women health professionals, health-seeking behavior of women, family support, and economic status were factors associated with knowledge and practice of antenatal exercise among women [5, 8]. Furthermore, history of abortion or infertility treatments, discomfort during exercise, and fear of injury to the fetus have all been mentioned by pregnant women as reasons for decreasing physical activity [16, 17].

Findings from few previous studies in Ethiopia were either from single institution which make them underpowered or only limited to hospital settings [18, 19]. Furthermore, information is scarce about the issue in the study setting. This study explored knowledge, attitude and practice of antenatal exercise in multi-center and with different tool than studies done before. So, the aim of the study was to assess prevalence of knowledge, attitude, and practice of physical exercise during pregnancy among women attending public health institutions in Hawassa city, Sidama in 2021.

## Methods

### Study area

The study was conducted in public health centers in Hawassa city, which is located 273 km from Addis Ababa, the capital city of Ethiopia. According to report of Hawassa city health department, the total population of town was estimated to be 369,908 in 2017 [20].

Hawassa has an elevation of 1,708 m above sea level. Administratively, the city is structured into 7 urban sub-cities and 21 Kebeles. Publically, there is 1 comprehensive specialized referral and teaching hospital, 1 public general hospital, 1 public primary hospital, 3 private primary hospitals, 11 public health centers, 1 private health centers, and 17 health posts. Antenatal care is provided in all of these health institutions.

### Study design and period

Facility based descriptive cross sectional study design was conducted in public health facilities in Hawassa city, Sidama, Ethiopia from July to December 2021.

### Population

#### Source population

All pregnant women who attended antepartum in public health institutions of Hawassa city were the target population in this study.

#### Study population

Pregnant women who attended antenatal care during the data collection period and presented at a randomly selected public health centers in Hawassa city during the study period were considered as study population.

### Eligibility criteria

All pregnant women who visited antenatal care follow up were included in this study. Pregnant women who came for medical or other purpose, pregnant mothers who had been diagnosed with gait disturbances, and serious psychiatric illness were excluded from this study.

### Sample size determination and procedure

Sample size was determined using single population proportion formula. The sample size was computed using the following assumptions: Proportion of attitude towards physical exercise among pregnant women from previous study conducted in Gondar (P = 55.3%) [15], 95% confidence interval, and 5% marginal error. Where, n = number of samples, Z α/2 = the value of under standard normal value of confidence (1.96), and α = level of significance. Standard Cochrane formula was used to compute sample size, n= (Z α/2)^2^ P (1-P)/d^2^).

Therefore, plugging these values into formula, n = (1.96)^2^ 0.55* (0.45)/ (0.05)^2^, the initial sample size was calculated to be 384. On the contrary, pregnant women in public health institutions in Hawassa city was reported as less than 10,000 (N = 1784) within the past 3 months before this study. Thus, a reduction formula (n = n/1 + n/N) have been applied making initial sample size of 313. Finally, we have added 10% non-response rate as the compensation for non-respondents making final sample size of 344.

All health centers were included in this study. Each pregnant woman was selected using systematic sampling technique during antepartum visits. Each K-value was determined by using formula (K = N/n). Therefore, based on the client flow in the selected health centers, we have used different K-values to select samples. The first study participant was chosen among clients came for antenatal care service using lottery method.

### Data collection method, tools and procedure

This descriptive cross-sectional study explored pregnant women knowledge, practice and attitude of pregnant women. Interview administered survey was applied to collect data in this study. Data were collected using semi-structured and pre-tested questionnaire. The tool was adapted from various literatures [18, 19, 21]. It includes information about socio-demographic, health care seeking, and reproductive health charactestics of the study subjects. Information on socio-demographic factors includes age, educational status, income and occupation. Obstetrics factors include number of birth, gestational age, mode of last delivery and history of abortion. Furthermore, these studies include attitude, practice and knowledge of physical exercise. Data were collected by 4 BSc midwives and supervised by two MSc clinical midwives. Overall supervision was conducted by principal investigator.

### Data quality control

Questionnaire was first prepared in English and translated to Amharic and Sidaamu Afoo (local languages) and re-translated back to English to keep its consistency. Pre-test was done on 18 (5%) study participants on outside of the study area (Shashemenie). Training was given for 4 data collectors and 2 supervisors for one day. Training was focused on objective of the study, data collection tool, and methods of data collection, checking the completeness questionnaires and maintains confidentiality. Two supervisors monitored data collection process. Questionnaire was checked for completeness before data entry. Any problem which was occurred during data collection was discussed and solved each day after data collection together with principal investigator and co-investigator.

### Data analysis

The purpose of analysis of this study was to describe the prevalence of knowledge, attitude and practice of physical exercise among pregnant women. Data were cleaned and entered in Epi-data version 4.6 and exported to SPSS version 25 for analysis. Descriptive statistics was performed using frequency, mean, standard deviation and percentage. Data was presented and reported using text, table, graph and figure.

### Ethical consideration

#### Ethical approval

and clearance was obtained from Hawassa University, college of medicine and health sciences, health Ethical Review Board/IRB/. The purpose of the study was explained for the study participants, and written informed consent was obtained. The right to withdraw from the study at any time was assured before the start of data collection. Coding was used to eliminate any personal identification. Confidentiality was assured throughout the study.

### Operational definitions

**Knowledge**: Women’s knowledge about physical exercise was measured based on the individual study participant’s correct response of 9items measuring their knowledge about exercise. Each question had one correct answer and three options: Yes, No and ‘I do not know’. Those who scored above the mean of the items are labeled as women with “adequate knowledge”, otherwise, ‘in adequate knowledge’.

#### Attitude

Attitude was measured using 8 questions with possible three responses. Those participants who scored above the mean were considered as having ‘positive attitude’ towards physical exercise, otherwise, they were considered as having ‘negative attitude’.

#### Practice

If pregnant women perform any type of antenatal physical exercise in the current pregnancy at least 3 times per week from a total of 6 exercises, they were regarded as doing ‘good practice’; otherwise, ‘poor practice’.

## Results

### Socio-demographic characteristics

All of 344 samples complete interview making a response rate of 100%. According to Table 1 report of this study, the mean age of the study subjects was estimated as 26.8 with SD ± 4.9 years.

**Table 1:** Socio-demographic characteristics of pregnant women (n = 344)

Variables		Frequency	Percent
Age	17–25 years	149	43.3
	26–34 years	165	48.0
	More than 35	30	8.7
Educational level	Below elementary	132	38.4
	High school	73	21.2
	Diploma and above	139	40.4
Marital status	Married	292	84.9
	Single	15	4.4
	Divorced	17	4.9
	Widowed	7	2.0
	Cohabitating	13	3.8
Religion	Orthodox	122	35.5
	Protestant	143	41.6
	Catholic	25	7.3
	Muslim	43	12.5
	Others	11	3.2
Occupational status	Gov. Employee	108	31.4
	House wife	91	26.5
	Private business	91	26.5
	Unemployed	54	15.7
Income Status	Less than 4500 ETB	116	58.3
	More than 4500 ETB	83	41.7

From the total of study subjects, majorities (84.9%) were married. One in five pregnant women (21.2%) has completed high school education. Thirty one point four (31.4%) of the study subjects were governmental employees; however, only one-quarter (26.5%) of study participant has already established their private business. Most (58.3%) of the study participants has monthly income level of less than 4,500 Ethiopian birr (**See** Table 1).

### Obstetric characteristics

Table 2 clearly describes the reproductive charactestics of study participants. Hence, most (76%) of the study participants have got more than one pregnancy. From the total study participants, 14% (14%) have had history of abortion. Besides, half of pregnant women were in the third trimester (32 weeks) of pregnancy (**See** Table 2).

**Table 2:** Obstetrical characteristics of pregnant women in Public health institutions of Hawassa city, Sidama Ethiopia, 2021 (n = 344)

Study variables	Category	Frequency	%
Parity	Null-parity	76	22.1
	Multi-parity	264	76.7
	Grand-multipara	4	1.2
Mode of last delivery	Normal	232	86.6
	Caesarean section	36	13.4
Gestational age	Less than 16 wks	69	20.1
	From 16 week to 32 weeks	119	34.6
	More than 32	156	45.3
History of abortion	Yes	48	14.0
	No	296	86.0

### Knowledge about physical exercise

In this study, prevalence of knowledge about physical exercise was computed as 42.2% (95% CI, 39.5%, 44.9%).

Table 3 presents knowledge of antenatal care exercise during pregnancy. From these components, most (63.1%) of study participants have never been counseled about physical exercises. Majorities (72.1%) of the study subjects knew that exercise can reduce excessive weight gain during antenatal care (**See** Table 3).

**Table 3:** Knowledge about the benefit of antenatal exercise among pregnant women attending prenatal care at public health centers in Hawassa city, Sidama, Ethiopia, 2021(n = 344)

Variable		Frequency	Percent
Reduce back pain	Yes	195	56.7
	No	32	9.3
	I do not know	117	34.0
Reduce excessive weight	Yes	248	72.1
	No	34	9.9
	I do not know	62	18.0
Strengthen pelvic floor	Yes	137	39.8
	No	72	20.9
	I do not know	135	39.2
Reduce risk of GDM	Yes	141	41.0
	No	71	20.6
	I do not know	132	38.4
Increase energy	Yes	183	53.2
	No	51	14.8
	I do not know	110	32.0
Rapid postnatal recovery	Yes	169	49.1
	No	55	16.0
	I do not know	120	34.9
Help to cope up with labor	Yes	156	45.3
	No	54	15.7
	I do not know	134	39.0

**Fig. 1:**
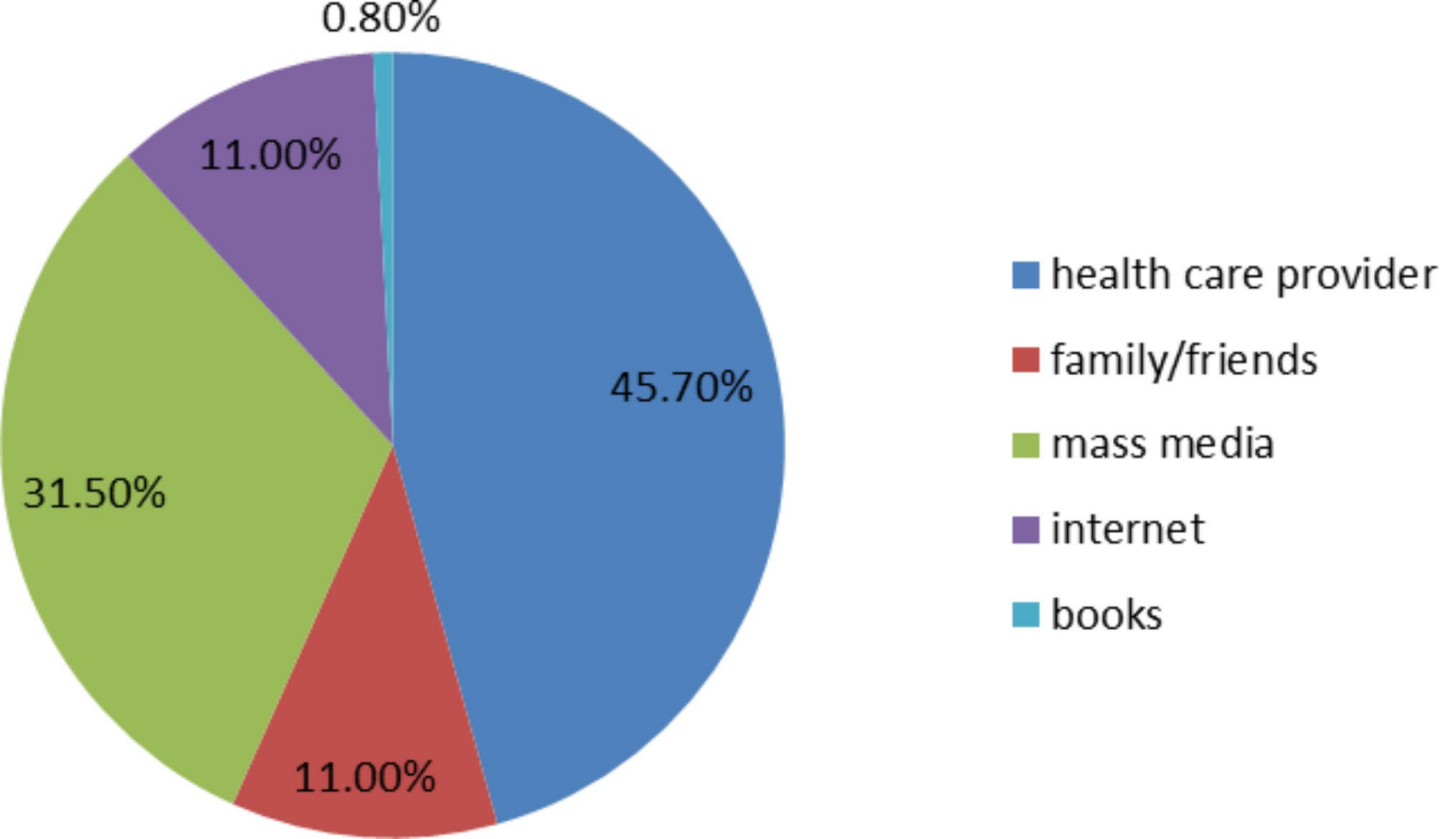
Source of information about physical exercise for pregnant women in Hawassa, Sidama Ethiopia

Figure 1 displayed pregnant women’s source of information about physical exercise. The commonly cited source of information were health care providers (45.7%) followed by mass media (31.5%) (**See** Fig. 1).

Two hundred thirty six (68.6%) of study participants have heard about physical exercise before pregnancy. Of these who heard about antenatal exercise, Majorities 216 (62.8%), 211(61.3%) were told about walking exercise. On the other hand, forty three point nine (43.9%) of the study participant were informed about the benefit of back care exercise during pregnancy (**See** Table 3).

### Knowledge about physical exercise during pregnancy

Based on the report of Table 4, most (72.1%) of the study participants have identified breathing difficulty as a contraindication for antenatal exercise during pregnancy. On the contrary, only 27.6% of the study participants clearly describe anaemia as contraindication for exercise during pregnancy. Anemia (56.1%), type I diabetic mellitus (54.4%) and vaginal bleeding (54.1%) were major medical complications which were not mentioned by study participants as a contraindication for exercise during pregnancy (**See** Table 4).

**Table 4:** Knowledge about the contra-indication of antenatal exercise among pregnant women attending antenatal care at public health centers in Hawassa city, Sidama, Ethiopia, 2021(n = 344)

Variable	Category	Frequency	Percent
Chest pain	Yes	137	39.8
	No	46	13.4
	I do not know	161	46.8
Breathing difficult	Yes	142	41.3
	No	48	14.0
	I do not know	154	44.8
Abdominal pain	Yes	114	33.1
	No	64	18.6
	I do not know	166	48.3
TYPE 1 DM	Yes	86	25.0
	No	71	20.6
	I do not know	187	54.4
During uterine contraction	Yes	112	32.6
	No	59	17.2
	I do not know	173	50.3
Vaginal bleeding	Yes	106	30.8
	No	52	15.1
	I do not know	186	54.1
Premature labor	Yes	117	34.0
	No	52	15.1
	I do not know	175	50.9
When fetal heart beat reduced	Yes	96	27.9
	No	65	18.9
	I do not know	183	53.2
Anemia	Yes	95	27.6
	No	56	16.3
	I do not know	193	56.1

### Attitude towards antenatal exercise

Table 5 highlights attitude of study participants about physical exercise during pregnancy. Generally, most 219 (63.7%) of the study participants had positive attitude towards antenatal exercise. About four out of five women (80.8%) perceived that physical exercise during pregnancy was essential in this study. However, only 23.8% study participants mentioned that culture does not suit exercise (**See** Table 5).

**Table 5:** Percentage distribution of attitude of antenatal exercise among pregnant women attending antenatal care at public health centers in Hawassa city, Sidama, Ethiopia, 2021 (n = 344)

Variable	Category	Frequency	Percent
Doing exercise is essential during pregnancy	Yes	278	80.8
	No	27	7.8
	I do not know	39	11.3
Reduce pregnancy related complication	Yes	220	64.0
	No	83	24.1
	I do not know	41	11.9
regular exercise facilitate normal delivery	Yes	246	71.5
	No	55	16.0
	I do not know	43	12.5
exercise will help recovery after delivery	Yes	209	60.8
	No	79	23.0
	I do not know	55	16.0
exercise is safe for the foetus	Yes	173	50.3
	No	121	35.2
	I do not know	50	14.5
exercise does not suit our culture	Yes	82	23.8
	No	230	66.9
	I do not know	32	9.3
Doing exercise without advice of health care professional	Yes	102	29.7
	No	203	59.0
	I do not know	39	11.3
Doing make energetic	Yes	182	52.9
	No	129	37.5
	I do not know	33	9.6

### Prevalence of practice of antenatal exercise

Overall, proportion of study participants who practiced antenatal exercises during pregnancy was calculated as 35.8%.

**Fig. 2:**
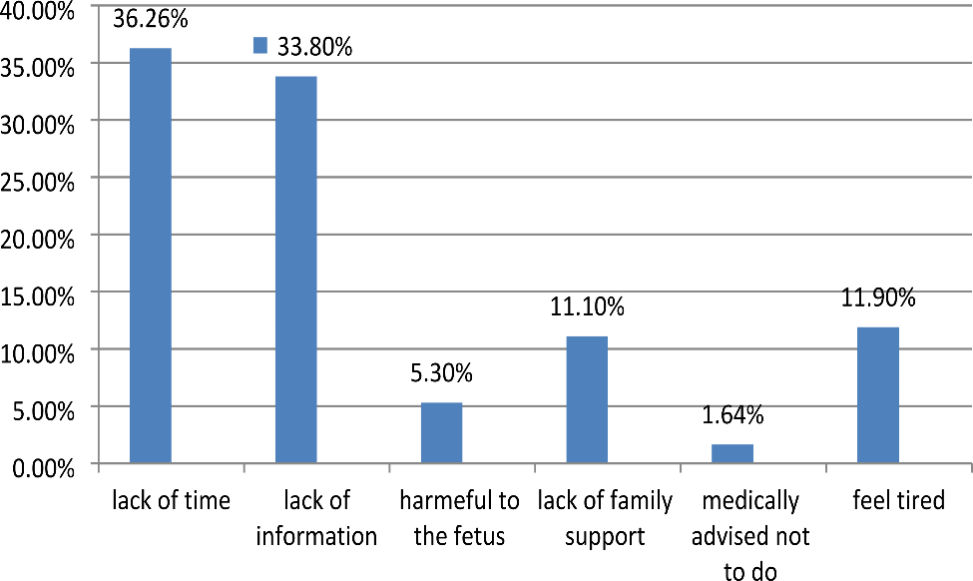
Reasons for not doing in the current pregnancy exercise among pregnant women attending prenatal at health centers in Hawassa city, Sidama, Ethiopia, 2021**(**n = 221)

Figure 2 presents the common reasons of the study participants’ failure to engage into physical exercise were expressed as lack of time; scarce information and tiredness feeling with value 36.3%, 33.8% and 11.9%, respectively (**See Fig. 2**).

**Fig. 3:**
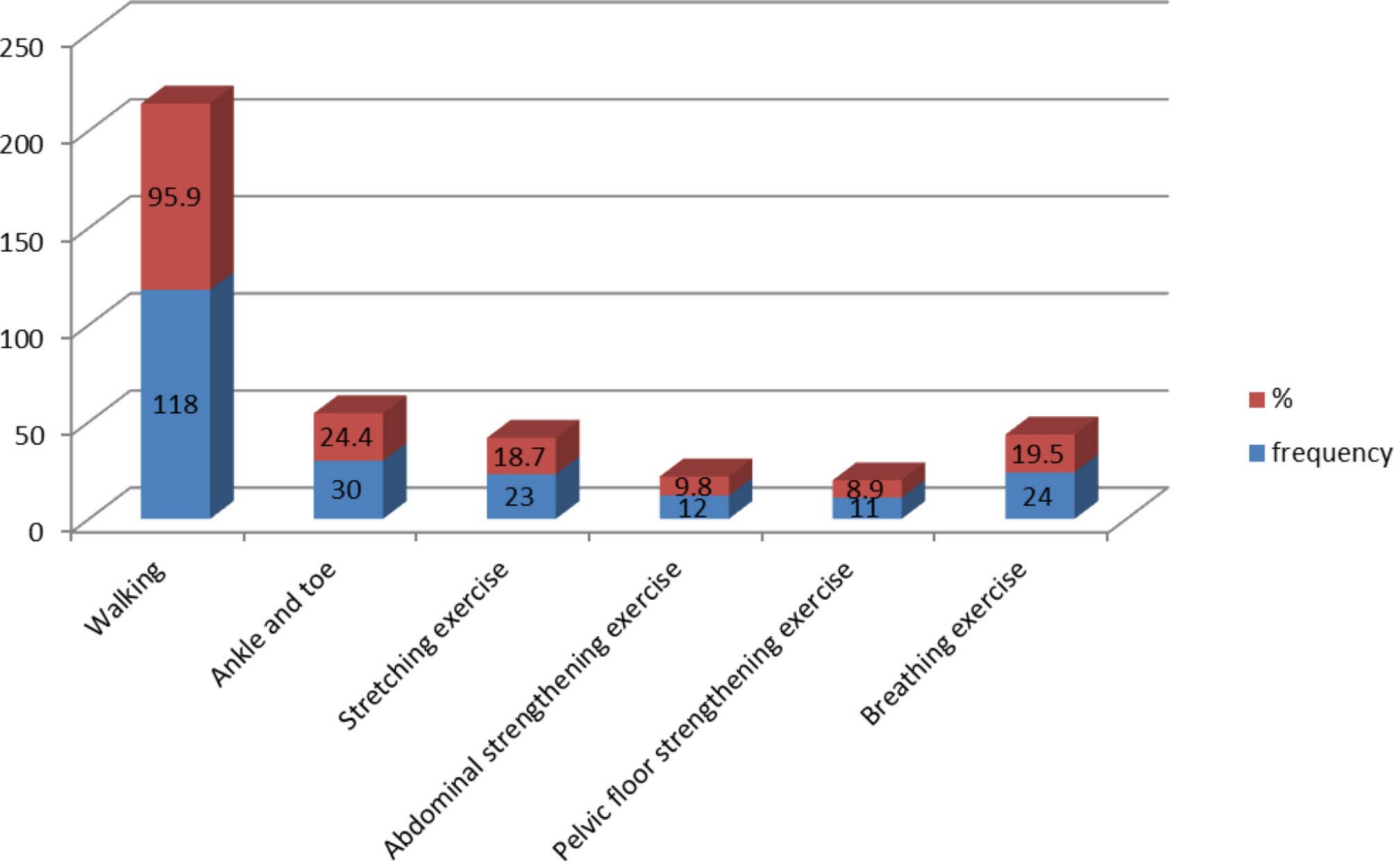
Types of physical exercise practices among pregnant women in Hawassa city Health centres, Sidama, Ethiopia (n = 344)

Figure 3 illustrates types of physical exercise practiced by pregnant women in the study area. For example, waking was practice by most (95.9%) of the study participants; however, pelvic floor exercise is the least (8.9%) component of physical exercise practiced among the study subjects **(See** Fig. 3).

## Discussion

The study was conducted to assess prevalence of knowledge, attitude and practice of antenatal exercise among pregnant women in Hawassa city, public health centers, Sidama region, Ethiopia. In this study, prevalence of knowledge of physical exercise among pregnant women was estimated as 42.2% (95% CI, 39.5-44.9%). The finding is consistent with finding of the study done in Gondar, Northwest, Ethiopia [18]. This could be explained by consistency of socio-demographic charactestics, study setting and health information access.

This finding is lower than finding of a study conducted in Mekelle (51%) [22], Addis Ababa (50.4%) [23] and Nigeria [24]. This inconsistency may be due to variation in the health system, unavailability of antenatal exercise guideline and antenatal care education class in the current study.

The finding of current study is higher than finding of study done in Zambia [25]. This discrepancy might be due variation in study participants’ educational status, experience and access to health information during antenatal care.

We have found out that magnitude of positive attitude towards physical exercise among pregnant women during pregnancy was computed as 64%( 95%CI: 61.4–66.6%). This finding is higher than finding of a study done in Gondar (55.3%) [18] and Addis Ababa (27.9%) [23]. The difference might be due to barrier in information dissemination about the benefit of exercise, local culture and religious of study participants. However, this finding is lower than finding of the study conducted in Pakistani [26]. The reason for difference might be difference in socio-demographic characteristics, cultural, access to health care and exposure to technology.

This study depict that practice of physical exercise was estimated as 35.8% in this study during their current pregnancy. This finding is higher than finding of studies done in most Ethiopian cities: Gondar (30.9%) [18] and Mekelle(16.6%) [22], Addis Ababa(22.3%) [23], Arbaminich(32.9%) [27] and Nigeria [28]. This difference might be due variation in awareness and experience of physical exercise before pregnancy. Furthermore, lack of motivation for counseling among health care providers might also be the possible explanation for the difference.

This study demonstrated that walking, ankle and toe exercise and breathing were the most common practiced, respectively. This finding is in agreement with finding of study conducted in Sirilanka [29]. The common reasons for pregnant women for failure of engaging in antenatal exercise in the current pregnancy were listed as lack of time (36.26%), lack of information (36.8%) and feel tired (11.9%). Those cited reasons are consistent with a studies ascertained from Brazil and Canada [29, 30]. Generally, this study findings shows that knowledge, attitude and practice of pregnant women are below the recommended guidelines [31, 32]. The possible rational might be due to lack of information, local traditions and belief, less access to counseling and lack of clear guideline for management regarding about exercise prepared for obstetrics care providers.

### Public health implication

#### Policy implication

Policy makers should focus on developing applicable guideline for physical exercise during pregnancy, training manual and integration of counseling session about physical exercise during antenatal care. Health facility managers should arrange conditions for practical physical exercise demonstration for health providers. Training should be provided for skilled care providers about benefit, indication and outcome of physical exercise during pregnancy. Future researchers should focus on analytical studies to examine factors associated with low KAP level and qualitative studies to explore perception and experience of women for physical exercise during pregnancy.

#### Practical implication

Health providers should strengthen awareness creation about physical exercise during pregnancy.

### Limitation of the study

This study describes comprehensive aspect of knowledge, attitude and practice of physical exercise among pregnant women in multi-center in the study area. It suffered from poor comparison due to difference in operational definitions and few studies.

## Conclusion

Although attitude and practice of physical exercise are relatively higher than other studies in Ethiopia, knowledge about physical exercise was lower than most studies done in Ethiopia.

However, Knowledge, attitude and practice of physical exercise are found to be unsatisfactory compared with international guidelines. Therefore, policy makers should develop contextual guidelines for physical exercise in Ethiopia. Counseling about physical exercise should be integrated in maternal health service clinics. Future researcher should focus on identification of factors related with KAP of physical exercise.

## Data Availability

For those who are interested; the datasets of this study could be accessed from the corresponding author on reasonable request.

## List of Abbreviations

ANC: Ante Natal Care
KAP: Knowledge Attitude Practice
SPSS: Statistical Package for Social Science
WHO: World Health Organization
GDM: Gestational Diabetes Mellitus
ACOG: American Congress of Obstetricians and Gynecologists
IRB: Institutional Review Board
